# Retinal changes in visceral leishmaniasis by retinal photography

**DOI:** 10.1186/1471-2334-14-527

**Published:** 2014-09-30

**Authors:** Richard James Maude, BUM Wahid Ahmed, Abu Hayat Md Waliur Rahman, Ridwanur Rahman, Mohammed Ishaque Majumder, Darryl Braganza Menezes, Abdullah Abu Sayeed, Laura Hughes, Thomas J MacGillivray, Shyamanga Borooah, Baljean Dhillon, Arjen M Dondorp, Mohammad Abul Faiz

**Affiliations:** Mahidol-Oxford Tropical Medicine Research Unit, Faculty of Tropical Medicine, Mahidol University, Bangkok, Thailand; Centre for Tropical Medicine, Nuffield Department of Medicine, University of Oxford, Oxford, UK; College of Medicine and Veterinary Medicine, University of Edinburgh, Edinburgh, UK; Sir Salimullah Medical College, Dhaka, Bangladesh; Queen Elizabeth Hospital, Birmingham, UK; Chittagong Medical College Hospital, Chittagong, Bangladesh; Clinical Research Imaging Centre. Queen’s Medical Research Institute, University of Edinburgh, Edinburgh, UK

**Keywords:** Leishmaniasis, Retina, Retinopathy, Visceral, Bangladesh, Kala-azar

## Abstract

**Background:**

In visceral leishmaniasis (VL), retinal changes have previously been noted but not described in detail and their clinical and pathological significance are unknown. A prospective observational study was undertaken in Mymensingh, Bangladesh aiming to describe in detail visible changes in the retina in unselected patients with VL.

**Methods:**

Patients underwent assessment of visual function, indirect and direct ophthalmoscopy and portable retinal photography. The photographs were assessed by masked observers including assessment for vessel tortuosity using a semi-automated system.

**Results:**

30 patients with VL were enrolled, of whom 6 (20%) had abnormalities. These included 5 with focal retinal whitening, 2 with cotton wool spots, 2 with haemorrhages, as well as increased vessel tortuosity. Visual function was preserved.

**Conclusions:**

These changes suggest a previously unrecognized retinal vasculopathy. An inflammatory aetiology is plausible such as a subclinical retinal vasculitis, possibly with altered local microvascular autoregulation, and warrants further investigation.

**Electronic supplementary material:**

The online version of this article (doi:10.1186/1471-2334-14-527) contains supplementary material, which is available to authorized users.

## Background

Visceral leishmaniasis (VL) is a systemic disease caused by parasites of the *Leishmania donovani* complex. It is a major cause of morbidity and mortality in the north of Bangladesh, as well as parts of India, Nepal, Sudan and Brazil.

There have been occasional reports of retinal lesions in VL but there has not been a detailed systematic study and their clinical and pathological significance are unknown. Findings to date have been predominantly large, superficial retinal haemorrhages [1–7] often associated with thrombocytopaenia and/or anaemia, although cytoid bodies, dilated veins and vascular sheathing [2] as well as tortuous veins, retinal oedema and peripheral pallor [3] have also been described.

A prospective observational study was undertaken using portable retinal photography in Bangladesh aiming to describe in detail visible changes in the retina in patients with visceral leishmaniasis.

## Methods

The study was conducted from 2010-2011 at the Community Based Medical College Hospital and Trishal Upazilla Health Complex, Mymensingh District, Bangladesh. Ethical approval was obtained from the Institutional Ethical Committee of Sir Salimullah Medical College, Mitford, Dhaka, Bangladesh.

Consecutive unselected patients with confirmed visceral leishmaniasis by microscopy of splenic aspirate and/or RK 39 rapid diagnostic test were recruited provided they gave written informed consent. Exclusion criteria were: patients unable or unwilling to co-operate with eye examination; contraindications to dilating eye drops, such as angle closure glaucoma; and patients with media opacities precluding fundal view.

On admission, a full history and examination were carried out. Blood samples were obtained for haemoglobin, haematocrit, parasitaemia, platelet count, white cell count, ESR and biochemistry.

Eye examination included pupillary reflex to light and accommodation, visual acuity by Snellen chart, colour vision by Ishihara plates, visual fields by confrontation as well as direct and indirect ophthalmoscopy. Ophthalmoscopy was performed following pupillary dilation (0.5%/1% tropicamide). In addition, all patients had digital fundus photography using a portable handheld retinal camera (Kowa Genesis D, Kowa, Japan). To obtain images including the peripheral retina, a minimum of nine overlapping photographs (corresponding to approximately 15 megapixels) were required from each retina. These photographs were stitched into composite images using Photoshop CS3 Extended software (Adobe, San José, CA, USA). Photographs were examined by 2 masked investigators (BD and SB) for any retinal abnormalities and differences resolved through consensus.

Blood vessel width and tortuosity were measured from the retinal images using VAMPIRE (Vessel Assessment and Measurement Platform for Images of the REtina; version 2.0, Universities of Edinburgh and Dundee, UK) [8]. The software applies a multi-scale, 2-D Gabor wavelet which transforms the fundus images to emphasize the appearance of vessels and a supervised pixel classification is guided to identify vessel pixels by a Bayesian classifier. A trained user then manually identifies regions of vessels for tortuosity or width measurements. For tortuosity, the user selects the path for a chosen vessel and the algorithm integrates axis curvature and vessel width [9]. To measure vessel width the software returns the size of the automatically generated vessel map perpendicular to an estimated vessel axis. Where images allowed, the major arcade vessels were sampled at one disc diameter, two disc diameters and three disc diameters from the optic disc edge. As normal ranges for the VAMPIRE parameters have not yet been established, retinal photographs from 30 healthy individuals recruited in a separate study were used for comparison. These photographs were taken using the same retinal camera and were processed using the exact same methodologies as the patients with leishmaniasis. These data will be published separately.

Drug treatment was with intramuscular paromomycin, oral miltefosine and/or intravenous liposomal amphotericin in accordance with local guidelines.

Statistical analysis was performed using GraphPad Prism 6 (GraphPad Software, Inc., USA) and Excel 2007 (Microsoft Corp., Redmond, WA, USA). The level of significance was *P* < 0.05. For normally distributed unpaired values, means were compared using t-test and for non-normally distributed unpaired values medians were compared using the Mann-Whitney U test. For paired non-normally distributed values, the Wilcoxon matched pairs signed rank test was used.

## Results

**Figure 1:**
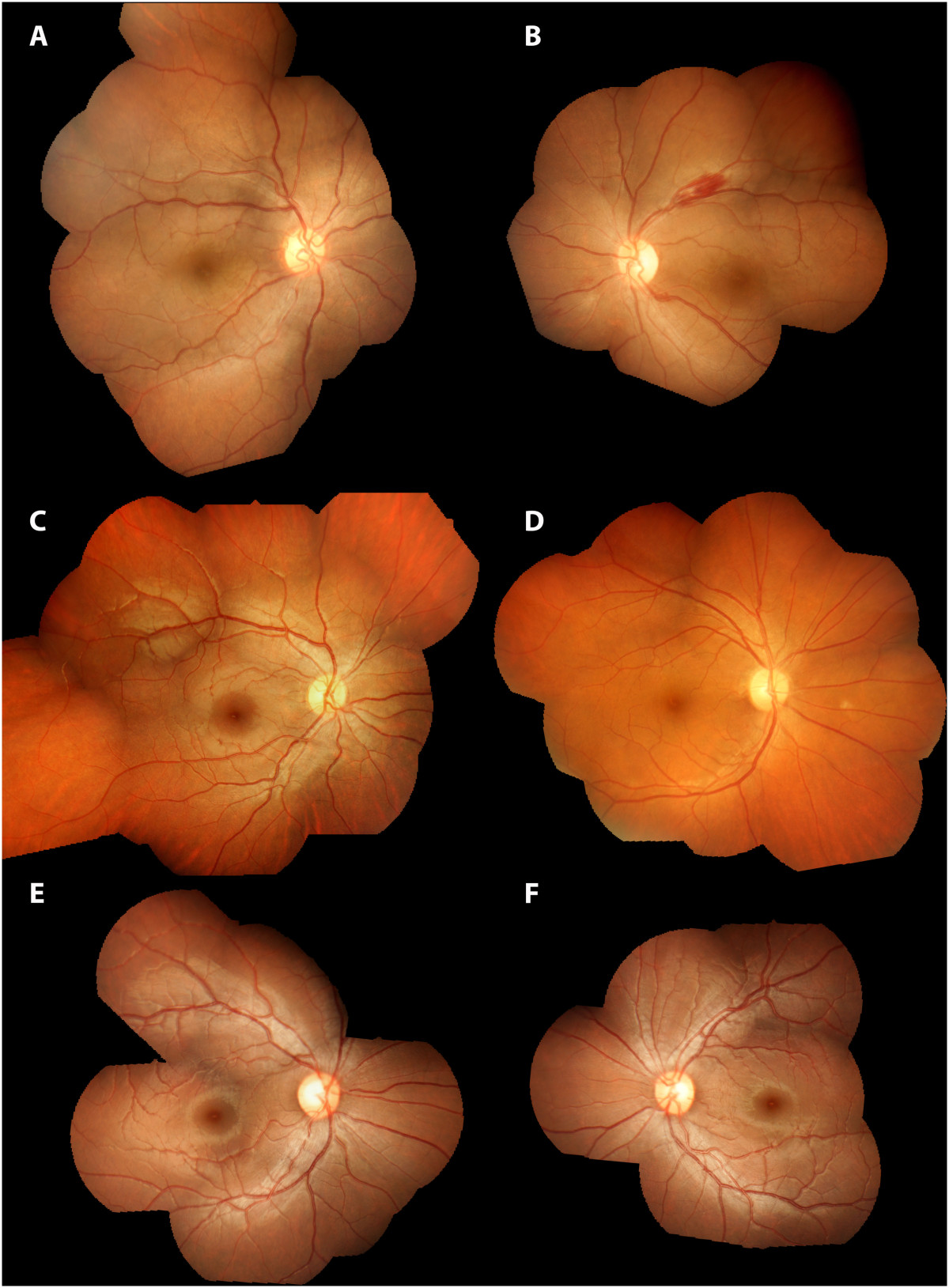
**Composite retinal photographs of patients with visceral leishmaniasis.** Details of retinal findings are listed in Table 2. **A** Patient 1 right eye, **B** Patient 1 left eye, **C** patient 3 right eye, **D** patient 4 right eye, **E** patient 2 right eye and **F** patient 2 left eye.

Thirty patients with visceral leishmaniasis were enrolled, 6 of whom had abnormal findings in the retina. No patients were excluded from the study and all had a minimum of 9 overlapping retinal photographs. The details of the enrolled patients are in Table 1 and the retinal findings in those with visible lesions are in Table 2. Findings included perivascular whitening, retinal haemorrhages and cotton wool spots. Examples of abnormalities seen on retinal photographs are in Figure 1. There were no differences in any of the criteria listed in Table 1 between those with retinal lesions and those without, except haemoglobin was lower in those with retinopathy (mean (95% confidence interval) of 7.5 (6.8-8.2) vs 9.7 (9.1-10.2) g/dL, p = 0.006. Colour vision, visual fields, pupil size and reaction to light were normal in all recruited patients. Median (IQR) visual acuity was 20/20 (20/20-20/53.5) with no difference between those with retinal lesions and those without.

**Table 1:** **Details of enrolled patients with visceral leishmaniasis**

Criteria	Value
Age (years)	23 (17-28)
Male sex, number (%)	17 (57%)
Hypertension, number (%)	1 (3%)
Diabetes mellitus, number (%)	0 (0%)
Systolic blood pressure (mmHg)	101 (96-105)
Lymphadenopathy, number (%)	3 (10%)
Hepatomegaly, number (%)	20 (67%)
Splenomegaly, number (%)	26 (87%)
PKDL, number (%)	0 (0%)
Positive RK39, number (%)	30 (100%)
Positive splenic aspirate, number (%)	22 (73%)
Haemoglobin (g/dL)	9.4 (8.8-9.9)
Leukocytes (cells/μL)	4.1 (3.5-9.9)
Platelets (cells/μL), median (IQR)	160 (112-190)
ESR (mm/h)	105 (88-121)
Prothrombin time (s), median (IQR)	14 (13-14)
Urea (mg/dL)	30 (27-33)
Creatinine (mg/dL)	1.0 (1.0-1.1)
Bilirubin (mg/dL), median (IQR)	0.5 (0.4-0.9)
Aspartate aminotransferase (U/L)	110 (38-182)
Alanine aminotransferase (U/L)	34 (23-96)
Glucose (mmol/L)	5.5 (4.2-6.8)

Numbers are mean (95% confidence interval) unless stated otherwise.

**Table 2:** **Retinal findings on retinal photography in the 6 patients with visible retinal lesions**

Patient	Eye	Haemorrhages	Cotton wool spots	Whitening	Vessels	Drusen
**1**	**R**	None	2 superotemporal	1 inferotemporal perivascular white dot	Normal	None
	**L**	1 superotemporal flame-shaped at first bifurcation	None	None	Tortuous, veins and arteries. Possible superotemporal vein occlusion at first bifurcation, inferotemporal vein occlusion at AV crossing first bifurcation	Few macular drusen
**2**	**R**	None	None	Perivascular	Temporal periarteriolar whitening/sheathing	None
	**L**	None	None	Perivascular	Normal	None
**3**	**R**	1 superficial	None	Perivascular	Normal	None
	**L**	None	None	Periarterial	Normal	None
**4**	**R**	None	1 nasal	Periarterial	Normal	None
	**L**	None	1 peripapillary	None	Normal	Macula, few
**5**	**R**	None	None	None	Normal	None
	**L**	None	None	None	Normal	None
**6**	**R**	None	None	None	Normal	None
	**L**	None	None	Multifocal	Normal	None

A medication which can affect the retina had been taken by 7/30 (23%) patients, 2 of whom had retinal lesions. These had all taken short courses (≤7 days) of oral ciprofloxacin which has rarely been associated with retinal detachment. None of the enrolled patients had retinal detachment. Previous eye disease was reported by three patients (dacryocystitis, congenital pigmented conjunctiva and vitamin A deficiency), none of whom had retinal lesions. None had a prior history of malaria. Visual symptoms reported prior to enrollment were blurred vision experienced by 4 individuals, one of whom had retinal lesions on photography (patient 1 in Table 2). As for neurological symptoms, 1 patient had seizures, 3 headache and 2 subjective weakness, all with normal neurological examination. Retinal abnormalities were seen in one of these patients with headache (patient 6 in Table 2).

### Tortuosity

Values were obtained for retinal vessel tortuosity and width for 14/30 patients with VL. The median (IQR) tortuosity in patients with VL (0.063 (0.02-0.17)) was greater than in healthy individuals (0.001 (0.001-0.003), p = <0.0001). There were no differences in tortuosity between veins and arteries or left and right eyes within individuals. Vessel widths were lower for retinal venules 2 optic disc diameters from the optic disc in VL than in healthy controls (mean (95% CI) 14.0 (12.0-15.9) vs 19.2 (18.3-20.1), p < 0.0001). There were no differences in the widths of venules 1 or >2 optic disc diameters from the disc or in arterioles.

## Discussion

A variety of novel, predominantly vascular, retinal lesions were seen in a fifth of patients with visceral leishmaniasis in this study. In the literature, reports of retinal findings in VL have been occasional patients mostly with retinal haemorrhages, although many of these were from a period when technology limited the ability to perform detailed retinal examination.

The predominant retinal changes in the present study were perivascular whitening and tortuous vessels. These findings would be consistent with a vasculopathy, perhaps a vasculitic process in the retina, possibly causing focal ischaemia. A small vessel vasculopathy affecting superficial vessels would explain the previously described superficial retinal haemorrhages and cotton wool spots also seen in this study. Another possible cause of increased tortuosity is raised intracranial pressure, although this is not thought to occur in VL. Overall, the pattern of retinal changes seen does not clearly fit for any known disease, including known causes of retinal vasculitis, and appear to be novel. It is possible that this represents a previously unrecognized specific retinopathy of VL.

Central nervous system involvement in VL is rarely reported and probably under-recognised [10]. Neuro-inflammation causing disruption of the blood brain barrier with transit of inflammatory cells and Leishmania amastigotes has been suggested as a possible mechanism [11]. In the eye, Leishmania amastigotes have been found in the aqueous humour in humans [12] and much more commonly and more widespread, including in the retina, in animals [11].

The abnormalities found in this study were predominantly vascular. A microvascular pathology has been previously described in affected organs in VL consisting of subendothelial oedema, hyalinosis and intima proliferation, possibly due to circulating immune complexes [13]. In addition, nitric oxide production is diminished *in vitro* in VL potentially impacting on microvascular function [14]. In dogs, VL causes a systemic vasculitis which can affect the eye, [15, 16] although retinal involvement has not been described. In humans, VL has occasionally been described in association with a variety of systemic vasculitides, including Wegener’s granulomatosis, [17, 18] polyarteritis nodosa, [19] Behćet’s disease, [20] mixed cryoglobulinaemia [21] and more general autoimmune activation [22] as well as vasculitis of the colon in a patient with HIV [23] although in at least some of these the VL is likely a secondary phenomenon following immunosuppression. The patients in this study had raised ESR, consistent with systemic inflammation but no other obvious manifestations of systemic autoimmune disease. Without clear evidence of a retinal ischaemic process in the present study, altered local microvascular autoregulation and a subclinical retinal vasculitis are possible contributors to the increased vessel tortuosity found.

This study had several limitations. Facilities for detailed examination and investigation of the patients was limited by available resources at the study sites. It was only possible to enroll a relatively small number of patients and a future study with a larger cohort would be more informative. It was not possible to definitively determine that the retinal abnormalities found are specific to VL. It is possible that there is another disease causing retinal lesions in the general population recruited in this study. To establish this, a much larger study would be required including photography of a range of patients with and without VL.

## Conclusions

The findings in this study clearly warrant further investigation. Although apparently common, it is fortunate that the retinal lesions seen do not appear to affect visual function. Whether they represent a novel retinopathy of VL and are evidence of a previously unrecognized vasculitic process occurring in the retina remains to be established. Additional techniques such as fluorescein angiography and optical coherence tomography would be particularly informative.

## Authors’ original submitted files for images

Below are the links to the authors’ original submitted files for images. Authors’ original file for figure 1

## Abbreviations

ESR: Erythrocyte sedimentation rate
HIV: Human immunodeficiency virus
VAMPIRE: Vessel Assessment and Measurement Platform for Images of the REtina
VL: Visceral leishmaniasis.

## References

[1] Acharyya C (1957). Retinal haemorrhage in kala-azar. J Indian Med Assoc.

[2] Tassman WS, O’Brien DD, Hahn K (1960). Retinal lesions in kala-azar. Am J Ophthalmol.

[3] Ling WP (1924). Ocular changes in kala-azar in Peking. Am J Ophthalmol.

[4] De Cock KM, Rees PH, Klauss V, Kasili EG, Kager PA, Schattenkerk JK (1982). Retinal hemorrhages in kala-azar. Am J Trop Med Hyg.

[5] Mookerjee GC, Sen G, Chaudhuri MD, Chakraborty K (1975). Acute kala-azar with haemorrhagic retinopathy. J Indian Med Assoc.

[6] Biswas J, Mani B, Bhende M (2000). Spontaneous resolution of bilateral macular haemorrhage in a patient with kala-azar. Eye (Lond).

[7] Montero JA, Ruiz-Moreno JM, Sanchis E (2003). Intraretinal hemorrhage associated with leishmaniasis. Ophthalmic Surg Lasers Imaging.

[8] Perez-Rovira A, MacGillivray T, Trucco E, Chin KS, Zutis K, Lupascu C, Tegolo D, Giachetti A, Wilson PJ, Doney A, Dhillon B (2011). VAMPIRE: Vessel assessment and measurement platform for images of the REtina. Conf Proc IEEE Eng Med Biol Soc.

[9] Trucco E, Azegrouz H, Dhillon B (2010). Modeling the tortuosity of retinal vessels: does caliber play a role?. IEEE Trans Biomed Eng.

[10] Hashim FA, Ahmed AE, el Hassan M, el Mubarak MH, Yagi H, Ibrahim EN, Ali MS (1995). Neurologic changes in visceral leishmaniasis. Am J Trop Med Hyg.

[11] Petersen CA, Greenlee MH (2011). Neurologic Manifestations of Leishmania spp. Infection. J Neuroparasitol.

[12] Ferrari TC, Guedes AC, Orefice F, Genaro O, Pinheiro SR, Marra MA, Silveira IL, Miranda MO (1990). Isolation of Leishmania sp. from aqueous humor of a patient with cutaneous disseminated leishmaniasis and bilateral iridocyclitis (preliminary report). Rev Inst Med Trop Sao Paulo.

[13] Veress B, el Hassan AM (1986). Vascular changes in human leishmaniasis: a light microscope and immunohistological study. Ann Trop Med Parasitol.

[14] Chowdhury KD, Sen G, Sarkar A, Biswas T (1810). Role of endothelial dysfunction in modulating the plasma redox homeostasis in visceral leishmaniasis. Biochim Biophys Acta.

[15] Garcia-Alonso M, Blanco A, Reina D, Serrano FJ, Alonso C, Nieto CG (1996). Immunopathology of the uveitis in canine leishmaniasis. Parasite Immunol.

[16] Pumarola M, Brevik L, Badiola J, Vargas A, Domingo M, Ferrer L (1991). Canine leishmaniasis associated with systemic vasculitis in two dogs. J Comp Pathol.

[17] Sollima S, Corbellino M, Piolini R, Calattini S, Imparato S, Antinori S (2004). Visceral leishmaniasis in a patient with Wegener’s granulomatosis. Rheumatology (Oxford).

[18] Zanaldi H, Rosenthal E, Marty P, Chichmanian RM, Pesce A, Cassuto JP (1999). Visceral leishmaniasis associated with Wegener disease. Use of lipid complex amphotericin B and liposomal amphotericin B. Presse Med.

[19] Scatena P, Messina F, Gori S, Ruocco L, Vignali C, Menichetti F, Castiglioni M (2003). Visceral leishmaniasis in a patient treated for polyarteritis nodosa. Clin Exp Rheumatol.

[20] Sirianni MC, Barbone B, Monarca B, Nanni M, Lagana B, Aiuti F (2001). A case of Behcet’s disease complicated by visceral Leishmaniasis and myelodysplasia: clinical considerations. Haematologica.

[21] Casato M, de Rosa FG, Pucillo LP, Ilardi I, di Vico B, Zorzin LR, Sorgi ML, Fiaschetti P, Coviello R, Lagana B, Fiorilli M (1999). Mixed cryoglobulinemia secondary to visceral Leishmaniasis. Arthritis Rheum.

[22] Wolga JI, Stahl JP, Gaillat J, Ribeiro CD, Micoud M (1983). [Immune and autoimmune manifestations of autochthonous visceral leishmaniasis with liver, kidney and vascular involvement]. Bull Soc Pathol Exot Filiales.

[23] Lemaistre AI, Chapel F, Cie P, Jeantils V, Guettier C (1997). [Unusual vascular lesions in the course of a colonic leishmaniasis in an HIV positive patient]. Ann Pathol.

[CR24] The pre-publication history for this paper can be accessed here:http://www.biomedcentral.com/1471-2334/14/527/prepub

