# Supplementary figures and images for: Retinal changes in visceral leishmaniasis by retinal photography
Source: BMC Infect Dis. 2014 Sep 30;14:527. doi: 10.1186/1471-2334-14-527 (PMC4261886; doi:10.1186/1471-2334-14-527)

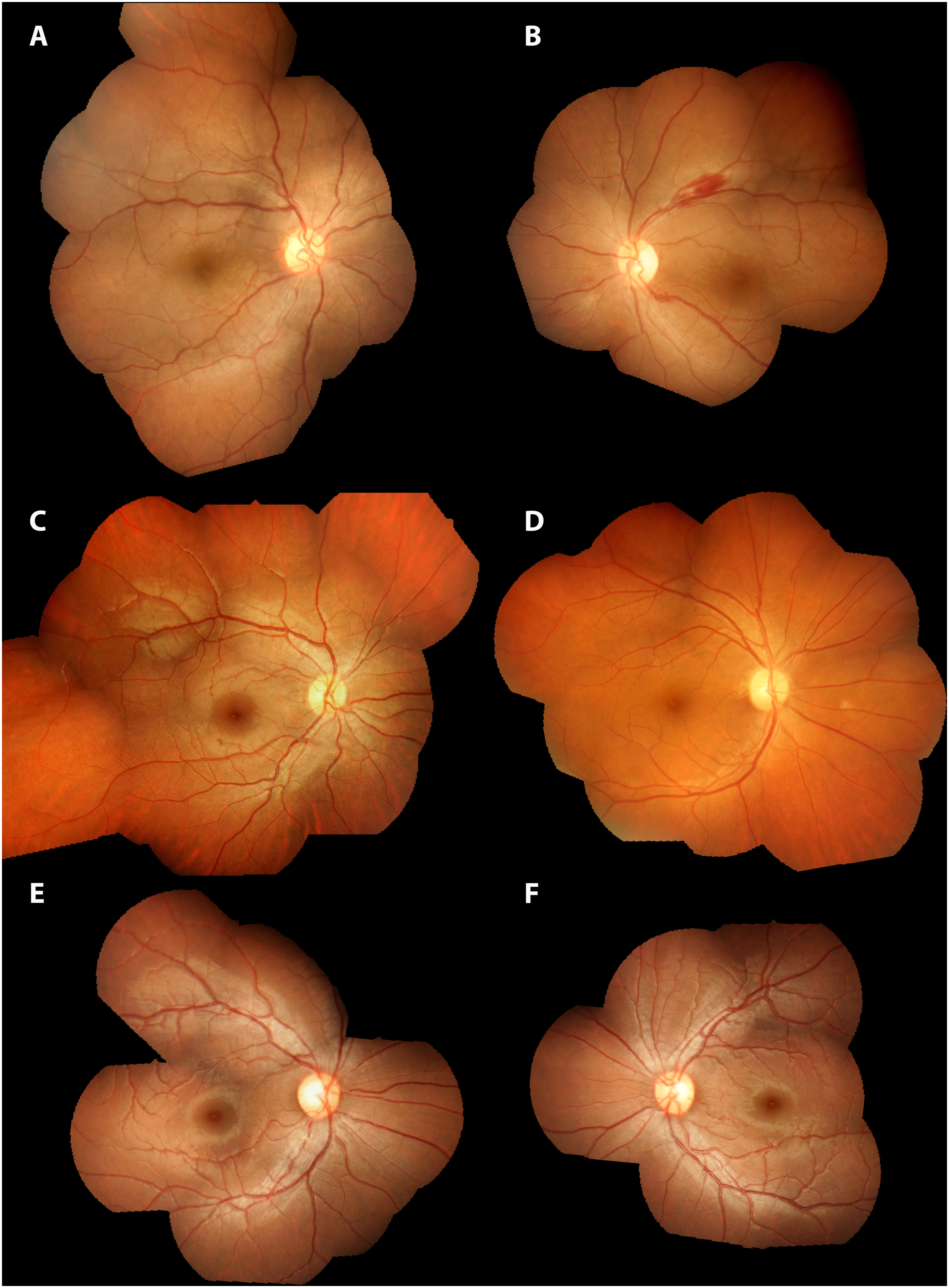

Supplement: Supplementary file 1 — Authors’ original file for figure 1 [file 12879_2014_3846_MOESM1_ESM.tif]
